# Filgrastim-Associated Pneumonitis in Cancer Patient Undergoing Hematopoietic Stem Cell (HSC) Mobilization for Autologous-HSC Transplantation

**DOI:** 10.7759/cureus.12114

**Published:** 2020-12-16

**Authors:** Drashti R Patel, Xavier Fonseca, Ashokakumar M Patel

**Affiliations:** 1 Pulmonary and Critical Care Medicine, Mayo Clinic, Rochester, USA

**Keywords:** filgrastim, granulocyte-colony stimulating factor, non-hodgkin’s lymphoma, autologous hematopoietic stem cell transplantation, bronchoalveolar lavage, thoracentesis

## Abstract

Filgrastim is a granulocyte-colony stimulating factors (G-CSF) used for multiple indications in cancer patients. We present a case of a 65-year-old man with non-Hodgkin’s lymphoma who was undergoing mobilization of hemopoietic stem cells for autologous-hematopoietic stem cell transplantation (auto-HSCT) with filgrastim who developed dyspnea and non-productive cough. Chest imaging showed left lower lobe consolidation, new ground-glass opacities and small right-sided pleural effusion. Bronchoscopy with bronchoalveolar lavage (BAL) and infectious evaluation were completely negative. He was admitted for further evaluation and management. Antibiotics weren’t started immediately given the clinical stability, multiple probable causes of fever and the intent of not confounding future thoracentesis results with antibiotic use. Thoracentesis occurred draining serous exudative pleural fluid; with follow-up chest imaging demonstrating no re-accumulation. His symptoms resolved and he was discharged in stable condition. The symptoms were hypothesized to be the probable adverse effects of filgrastim. We suggest close monitoring of pulmonary toxicities while administering this drug to patients to minimize such complications.

## Introduction

Autologous-hematopoietic stem cell transplantation (auto-HSCT) is the procedure of extraction and infusion of progenitor cells collected from the patient’s own marrow or peripheral blood, as opposed to cells from a donor, to repopulate or replace the recipient’s hematopoietic system. It is indicated in many disorders with depleted and dysfunctional bone marrow including many malignant and non-malignant disorders [1]. It acts as a salvage therapy for high dose chemotherapy in the management of malignancies, particularly hematologic by reducing post-chemotherapy pancytopenia and its complications [2]. Clinical studies have shown good results with the use of recombinant hematopoietic growth factors like granulocyte-colony stimulating factor (G-CSF) and granulocyte macrophage-colony stimulating factor (GM-CSF) for mobilization of peripheral blood progenitor cells (PBPCs) or following auto-HSCT [3-6].^ ^Filgrastim is one of the G-CSFs used for this purpose and has a good safety profile.

Like any other treatment modality, HSCT is associated with some complications. These are divided into early (within the first 30 days of transplantation) or late (more than one month after transplantation). Significant pulmonary complications are a leading cause of morbidity and mortality following HCT [7]. But these have been attributed mostly to infections in the setting of neutropenia and pulmonary toxicity of chemotherapeutic drugs itself.

However, in our case, the likely cause of pneumonitis and pleural effusion is hypothesized to be filgrastim. Only a few studies in the past have deliberated on the adverse effect of this drug.

## Case presentation

A 65-year-old man with primary refractory Germinal center B-type likely diffuse large B-cell lymphoma (DLBCL) transformed from follicular lymphoma initially diagnosed in October 2019 was currently undergoing preparation for autologous stem cell transplant. He was status post multiple rounds of chemotherapy with BR (Bendamustine-Rituximab), R-CHOP (Rituximab-Cyclophosphamide, Doxorubicin, Vincristine, Prednisone) and R-ICE (Rituximab-Ifosamide, Carboplatin, Etoposide) and radiation with evidence of complete metabolic response on PET CT dated December 2019. He had stem cell stimulation and mobilization daily with filgrastim for four days in February 2020. By the fifth day, the patient had completed stem cell collection. He developed dyspnea and non-productive cough, and was subsequently admitted for further workup of infectious etiology in the setting of HSC mobilization for stem cell transplant. 

On the third day of stem cell stimulation and mobilization, he developed a right upper extremity swelling and the peripherally inserted central catheter line was removed. An ultrasound of the upper extremity veins was obtained the next day, which showed an acute deep venous thrombosis (DVT) in the right subclavian and axillary veins, extending into brachial veins. He was started on anticoagulation with enoxaparin twice daily for a minimum of three months of anticoagulation. Patient noted some dyspnea on exertion and mild non-productive cough that seemed to coincide with the initiation of filgrastim. However, in the setting of his acute DVT, a CT chest angiogram was obtained on the fifth day which was negative for acute pulmonary embolism (PE) but demonstrated bilateral new ground-glass opacities and left lower lobe consolidation which was presumed to be due to an infectious/inflammatory etiology. The CT also showed small right pleural effusion with associated atelectasis and small left pleural effusion.

He was subsequently seen by his pulmonologist as an outpatient on day 6 who recommended bronchoscopy with bronchoalveolar lavage (BAL) to look for the etiology of infiltrate and a diagnostic thoracentesis. The patient continued anticoagulation with enoxaparin and BAL was done on day 7, but because of the anticoagulation, thoracentesis could not be performed. He was admitted for further evaluation and management of continued dyspnea and cough. BAL appearance was slightly bloody, with a cell count of 318 nucleated cells and neutrophil predominance. Evaluation for infectious etiology was completely negative, including Nocardia, Influenza, RSV, Legionella, PCP, fungal smear, and gram stain.

His vitals during his initial hospital course were within normal limits with the exception of sinus tachycardia (110s-120s) and fever (38.6^o^C) responsive to acetaminophen. Soon after admission, he desaturated and required 3 L/min oxygen therapy via nasal cannula.

Initial lactate was 1.0 (Ref: 0.5-2.2 mmol/L). Blood cultures previously obtained were negative. Antibiotics were not started immediately given clinical stability, multiple probable non-infectious causes of fever (atelectasis, lymphoma, DVT, recent BAL), and the intent of not confounding future thoracentesis results with antibiotic use. Thoracentesis occurred on day 8, draining 1.2 L of serous exudative pleural fluid; with follow-up chest imaging demonstrating no re-accumulation of fluid in pleural space. Overnight oximetry revealed episodes of desaturations, and he was started on nocturnal 1 L/min supplemental oxygen. His symptoms resolved and his overall condition improved, and was discharged on day 9 in stable condition. 

## Discussion

Filgrastim is one of the G-CSFs approved for multiple indications in cancer patients [8,9]. It can be used to prevent post-chemotherapy neutropenia and its complications, reduce the severity and duration of said neutropenia, and for mobilization of hemopoietic stem cells from the bone marrow into peripheral circulation for collection via leukapheresis as one of the steps in auto-HSCT [10]. Our case represents the last indication. It is a relatively safe and well-tolerated drug. Its major adverse effects in auto-HCST recipients are musculoskeletal symptoms, anemia and thrombocytopenia. Pulmonary complications after HSCT are usually attributed to infections in the setting of neutropenia [7] and pulmonary toxicity of chemotherapy drugs itself [11].

Our patient’s respiratory symptoms seemed to coincide with the administration of filgrastim. There aren’t many published cases of this adverse effect of this drug. A previous case report by Vilaplana et al. [12] depicts a similar finding of respiratory failure and pleural effusion with filgrastim administration which was treated with supplemental oxygen, empiric antibiotics and high dose hydrocortisone.

A systematic review by Azolulay et al. [11] found that G-CSF induced pulmonary toxicity has a higher incidence (73 out of 84 cases studied; 86.9%) in cancer patients who had undergone three or more rounds of chemotherapy, suggesting that G-CSF increases sequestration and adhesion of neutrophils in the lungs leading to increased toxicity of neutrophil products on pulmonary endothelial and epithelial cells which were previously damaged by repeated chemotherapeutic drugs. Most of these patients developed serious pulmonary complications like ARDS, interstitial pneumonia and pleural effusion and had high mortality (24.6%). Our patient had undergone multiple rounds of chemotherapy with multiple drugs which could have damaged the pulmonary endothelial cells. However, two cases in the review [11] developed ARDS with the use of G-CSF alone suggesting its ability to cause pulmonary toxicity by itself.

**Figure 1:**
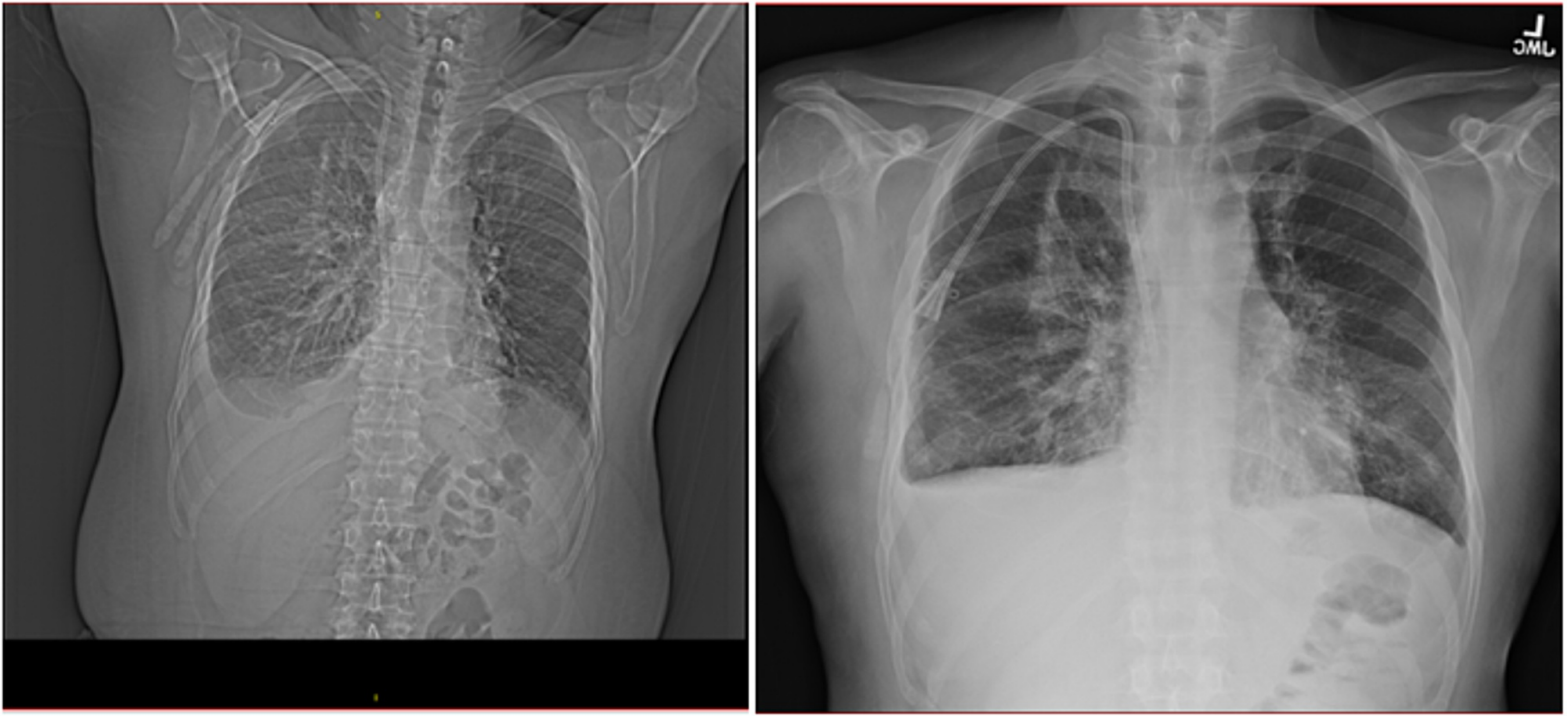
Chest CT scan: day 5 versus day 9.

Currently, there is no specific treatment for G-CSF toxicity [13] and the management is mostly symptomatic and supportive. A study by Karlin et al. [14] showed 20 cases of non-cardiogenic respiratory failure during G-CSF induced neutropenia recovery period. This emphasizes the severity of this adverse drug reaction as it can lead to acute respiratory distress syndrome which is associated with high mortality rates. Early diagnosis based on high suspicion and prompt treatment with immediate discontinuation of filgrastim is necessary, and mechanical ventilatory support when necessary. In the study by Karlin et al. [14], 80% of the patients needed mechanical ventilation. However, in our patient, oxygen therapy via high-flow nasal cannula was sufficient for the maintenance of oxygen saturation in the normal range during the day and didn’t require mechanical ventilation. The chest CT scan upon discharge (hospital day 9) showed no re-accumulation of fluid in pleural space, as shown in Figure 1, supporting our hypothesis of filgrastim induced pulmonary toxicity. The patient will be followed in Oncology clinic and Pulmonary outpatient clinic.

## Conclusions

Our report describes a patient who developed pneumonitis and pleural effusion as a probable adverse effect of filgrastim. Since there have been only a few published reports on the pulmonary toxicity of G-CSFs, we believe there is a need for more clinical research to identify the risk factors and exact mechanism of toxicity. We suggest close monitoring of pulmonary toxicities while administering this drug to patients to minimize such complications.

## References

[1] Khaddour K, Hana CK, Mewawalla P Hematopoietic stem cell transplantation- the future.

[2] Vaughan W, Seshadri T, Bridges M, Keating A (2009). The principles and overview of autologous hematopoietic stem cell transplantation. Cancer Treat Res.

[3] Smith TJ, Bohlke K, Lyman GH (2015). Recommendations for the use of WBC growth factors: American Society of Clinical Oncology Clinical Practice Guideline Update. J Clin Oncol.

[4] Siena S, Bregni M, Brando B, Ravagnani F, Bonadonna G, Gianni AM (1989). Circulation of CD34+ hematopoietic stem cells in the peripheral blood of high-dose cyclophosphamide-treated patients: enhancement by intravenous recombinant human granulocyte-macrophage colony-stimulating factor. Blood.

[5] Socinski MA, Cannistra SA, Elias A, Antman KH, Schnipper L, Griffin JD (1988). Granulocyte-macrophage colony stimulating factor expands the circulating haemopoietic progenitor cell compartment in man. Lancet.

[6] Sheridan WP, Begley CG, Juttner CA (1992). Effect of peripheral-blood progenitor cells mobilised by filgrastim (G-CSF) on platelet recovery after high-dose chemotherapy. Lancet.

[7] Afessa B, Abdulai RM, Kremers WK, Hogan WJ, Litzow MR, Peters SG (2012). Risk factors and outcome of pulmonary complications after autologous hematopoietic stem cell transplant. Chest J.

[8] Klastersky J, de Naurois J, Rolston K (2016). Management of febrile neutropaenia: ESMO Clinical Practice Guidelines. Ann Oncol.

[9] Ozer H, Armitage JO, Bennett CL (2000). 2000 update of recommendations for the use of hematopoietic colony-stimulating factors: evidence-based, clinical practice guidelines. J Clin Oncol.

[10] (2020). Neupogen - FDA. https://www.accessdata.fda.gov/drugsatfda_docs/label/2015/103353s5184lbl.pdf.

[11] Azoulay E, Attalah H, Harf A, Schlemmer B, Delclaux C (2001). Granulocyte colonystimulating factor or neutrophil-induced pulmonary toxicity: myth or reality?. Chest J.

[12] Vilaplana BE, Gutiérrez JR, de Ibarguen BC, Manrique MM, Guerrero AS (2016). Critically ill patient due to pneumonitis secondary to the use of filgrastim. Am J Ther.

[13] (2020). Neupogen - Medscape. https://reference.medscape.com/drug/g-csf-neupogen-filgrastim-342164#5.

[14] Karlin L, Darmon M, Thiéry G (2005). Respiratory status deterioration during G-CSF-induced neutropenia recovery. Bone Marrow Transplant.

